# Cholelithiasis and biliary sludge in Down's syndrome patients

**DOI:** 10.1590/S1516-31802007000600005

**Published:** 2007-11-01

**Authors:** Márcia Cristina Bastos Boëchat, Kátia Silveira da Silva, Juan Clinton Llerena Jr, Paulo Roberto Mafra Boëchat

**Affiliations:** Instituto Fernandes Figueira, Fundação Oswaldo Cruz, Rio de Janeiro, Rio de Janeiro, Brazil.

**Keywords:** Down syndrome, Gallbladder, Lithiasis, Cholecystitis, Cholecystectomy, Síndrome de Down, Vesícula biliar, Litíase, Colecistite, Colecistectomia

## Abstract

**CONTEXT AND OBJECTIVE:**

Although studies have demonstrated increased frequency of gallbladder abnormalities among Down's syndrome (DS) patients in some countries, there is only one paper on this subject in the Brazilian literature. The aim of this study was to demonstrate the prevalence, clinical characteristics and evolution of lithiasis and biliary sludge among DS patients in a maternity and children's hospital in Rio de Janeiro.

**DESIGN AND SETTING:**

This was a cross-sectional study followed by a retrospective cohort study on all individuals with an ultrasound diagnosis of gallbladder abnormalities.

**METHODS:**

547 DS patients (53.2% male, 46.8% female) attending the Instituto Fernandes Figueira in 2001 underwent abdominal ultrasound examination at ages of between one day and three years (mean: five months). Clinical and ultrasound data were analyzed.

**RESULTS:**

In 50 patients (9.1%), the ultrasound demonstrated gallbladder abnormalities (6.9% lithiasis and 2.1% biliary sludge). Spontaneous resolution was observed in 66.7% of the patients with biliary sludge and 28.9% with lithiasis. Cholecystectomy was carried out on 26.3% of the patients with gallstones.

**CONCLUSION:**

The results from this study and comparison with the literature suggest that DS patients are at risk of developing lithiasis and biliary sludge and should be monitored throughout the neonatal period, even if there are no known risk factors for gallstone formation. Most frequently, these gallbladder abnormalities occur without symptoms and spontaneously resolve in most non-symptomatic patients. DS patients should be monitored with serial abdominal ultrasound, and cholecystectomy is indicated for symptomatic cases or when cholecystitis is present.

## RESUMEN

**OBJETIVO:**

Demonstrar prevalência, características clínicas e evolução de litíase e lama biliar em pacientes com síndrome de Down (SD) num hospital materno-infantil no Rio de Janeiro. Apesar de estudos revelarem aumento das anormalidades biliares em pacientes com SD em alguns países, no Brasil existe apenas um trabalho abordando o tema.

**TIPO DE ESTUDO E LOCAL:**

Estudo transversal seguido por um estudo de coorte retrospectivo de todos os indivíduos com diagnóstico ultra-sonográfico de anormalidades da vesícula biliar.

**MÉTODOS:**

Foram selecionados 547 pacientes com SD (53,2% sexo masculino, 46,8% feminino) atendidos no Instituto Fernandes Figueira, Fundação Instituto Oswaldo Cruz (IFF-Fiocruz) em 2001. Todos os pacientes incluídos neste estudo foram submetidos a ultra-sonografia abdominal quando tinham idades variando entre um dia e três anos (mediana cinco meses). Dados clínicos e ultra-sonográficos foram avaliados.

**RESULTADOS:**

Em 50 (9,1%) crianças, a ultra-sonografia demonstrou alteração da vesícula biliar (6,9% litíase e 2,2 % lama biliar). Houve resolução espontânea em 66,7% dos pacientes com lama biliar e em 28,9% dos pacientes com litíase. A colecistectomia foi realizada em 26,3% dos pacientes com cálculo biliar.

**CONCLUSÃO:**

Resultados deste estudo e a comparação com a literatura sugerem que a SD deve ser considerada como fator de risco para desenvolvimento de litíase e lama biliar em crianças, sobretudo no período neonatal, sem que existam outros fatores predisponentes para formação de cálculo biliar. Na maioria das vezes, estas alterações são assintomáticas e, freqüentemente, têm evolução favorável, permanecendo desta forma ou tendo resolução espontânea. Pacientes devem ser acompanhados com ultra-sonografias seriadas. Tratamento cirúrgico está indicado para casos sintomáticos ou na presença de colecistite.

## INTRODUCTION

Before the 1980s, few cases of cholelithiasis in children, relating to hemolytic anemia, had been described in the medical literature.^1-3^ With the increased use of abdominal ultrasound, cholelithiasis has been diagnosed more frequently in children, and the reported prevalence is between 0.13% and 0.5% in different series.^4-7^ Several conditions are considered to be risk factors for biliary lithiasis among neonates, infants and children: hemolytic disease, cystic fibrosis, ileal resection, hypercholesterolemia, congenital hepatobiliary anomalies, congenital heart disease, prematurity, phototherapy, sepsis, parenteral nutrition, diuretics and antibiotics, particularly ceftriaxone.^8-11^

Some reports^12-16^ have showed that biliary abnormalities like lithiasis and biliary sludge occur more frequently among Down's syndrome (DS)* patients. However, only one of these reports^15^ relates to Brazilian patients.

## OBJECTIVE

The objective of this study was to determine the prevalence, clinical characteristics and evolution of biliary abnormalities in a group of 547 DS patients who were followed up at a maternity and children's hospital in the City of Rio de Janeiro, Brazil.

## MATERIAL AND METHODS

The patients in this study came from the register of 547 DS children who attended the Department of Medical Genetics of Instituto Fernandes Figueira, Fundação Oswaldo Cruz (IFF-Fiocruz) in the year 2001. All of these children underwent routine abdominal ultrasound when they began their treatment at the institute. Their ages ranged from one day to three years (mean: five months). At the time of the first ultrasound examination, all the patients were non-symptomatic and did not present any known risk factors for gallstones or biliary sludge.

For each patient, the following items were reviewed: sex, presence or absence of biliary abnormalities, age at the time of diagnosing the biliary abnormality on ultrasound, biliary abnormality type, clinical symptoms, other associated congenital anomalies, associated risk factors, type of treatment and clinical follow-up. All the individuals with an ultrasound diagnosis of gallbladder abnormality were monitored by abdominal ultrasound every three to six months during their first year and every year thereafter. All the patients were seen and followed up by the Departments of Medical Genetics and Pediatric Surgery.

**Figure 1:**
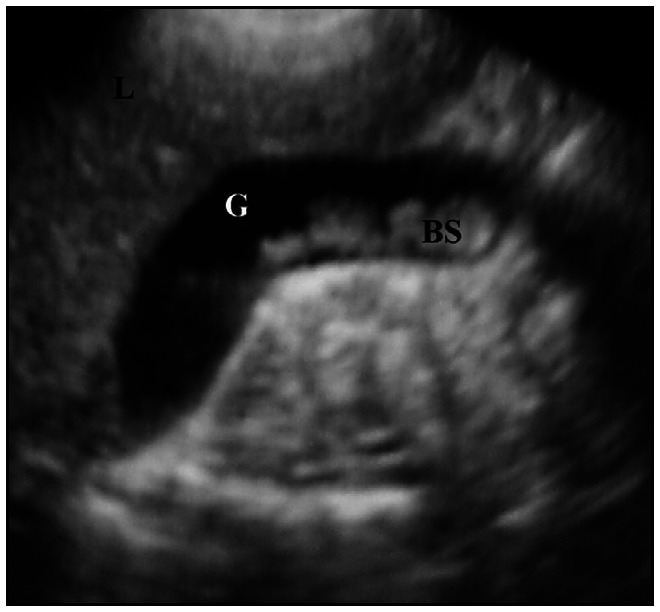
Ultrasound appearance of biliary sludge. G = Gallbladder, BS = Biliary sludge, L = Liver.

**Figure 2:**
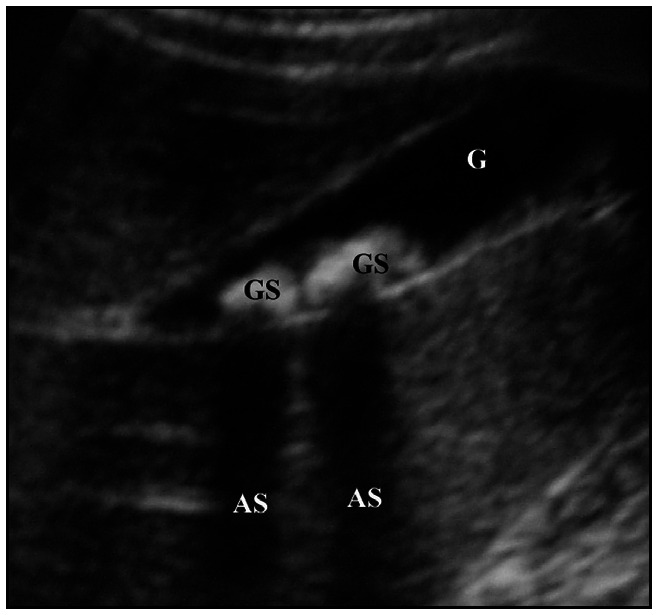
Ultrasound appearance of biliary lithiasis. G = Gallbladder, GS = Gallstones, AS = Acoustic shadow.

Ultrasound examinations were carried by means of a convex transducer (5 or 7 MHz) after the patients had been fasting for three to six hours. If after this period of time the gallbladder was not seen, the child remained without food for a further few hours until the examination could be adequately performed. The ultrasound diagnosis of biliary sludge and lithiasis was confirmed by parameters that are well-established in the literature.^17-18^ Echogenic material without an acoustic shadow in the gallbladder was interpreted as biliary sludge (Figure 1), while echogenic material with an acoustic shadow was considered to be a gallstone (Figure 2).

This project received approval from the institution's Ethical Review Board, under the number CAEE 0026.0.008.000-03 (Brazil). The EpiInfo 3.2 software (February 2004; Centers for Disease Control and Prevention, CDC, United States) was used to create a database and for all statistical analyses.

## RESULTS

Abdominal ultrasound examinations were performed before the age of two years in 94% of the patients. Ultrasound biliary abnormalities were identified in 50 individuals (9.1%): 38 patients had biliary lithiasis and 12 presented biliary sludge, with prevalences of 6.9% (95% confidence interval, CI: 5.1 - 9.6) and 1.7% (95% CI: 1.2 – 3.9), respectively. Table 1 describes the prevalence of these biliary abnormalities according to sex and age at the time of the ultrasound diagnosis.

**Table 1:** Prevalence of lithiasis and biliary sludge according to sex and the age at the time of ultrasound diagnosis

		Prevalence	
Age range in months	Normal (n = 497)	Lithiasis (n = 38)	Biliary sludge (n = 12)
	% (n)	% (n)	% (n)
0-2 (n = 135)	88.9% (120)	9.6% (13)	1.5% (2)
3-6 (n = 181)	91.1% (165)	5.0% (9)	3.9% (7)
7-12 (n = 113)	95.6% (108)	3.5% (4)	0.9% (1)
13-24 (n = 79)	91.1% (72)	6.3% (5)	2.5% (2)
25-36 (n = 39)	82.0% (32)	17.9% (7)	
**Sex**			
Female (n = 256)	46.9% (233)	42.1% (16)	58.3% (7)
Male (n = 291)	53.1% (264)	57.9% (22)	41.7% (5)

The ages of the DS patients with biliary sludge ranged from one month to two years (mean: 5.7 months), with slight predominance in females (58.3%). Spontaneous resolution of the sludge was observed in 66.7% of these patients and the others continued to present non-symptomatic biliary sludge for a period of three years, which was monitored by serial abdominal ultrasound examinations.

The ages of the DS patients with gallstones ranged from one day to three years (mean: nine months) at the time of the ultrasound diagnosis, with slight predominance in males (57.9%). Spontaneous resolution of the calculi occurred in 11 children (28.9%), over a period of three months to three years after the diagnosis (mean: nine months). Cholecystectomy was carried out on 26.3% of the patients with a mean age of 45 months; four of these patients were non-symptomatic and the other six were symptomatic. This surgery was indicated after a period of clinical and ultrasound follow-up (mean: 28 months). Eleven patients presented spontaneous resolution of their calculi and did not need to undergo operations. Among the remaining 27 patients, ten underwent operations (four without symptoms and six with symptoms), while 17 continued to present calculi. These 17 patients with lithiasis (44.7%) remained non-symptomatic for a longer period (mean: 45 months). Table 2 summarizes the main characteristics of the patients with biliary sludge and lithiasis.

**Table 2:** General characteristics of Down's syndrome patients with ultrasound diagnosis of lithiasis or biliary sludge

General characteristics		Lithiasis (n = 38)	Biliary sludge (n = 12)
**Sex**		
Male	22 (57.9%)	5 (41.7%)
Female	16 (42.1%)	7 (58.3%)
**Age at the time of ultrasound diagnosis**	1 day to 3 years (Mean: 9 months)	1 month to 2 years (Mean: 5.7 month)
**Symptoms**		
Non-symptomatic	32 (84.2%)	
Symptomatic	6 (15.8%)	12 (100%)
Abdominal pain	3 (7.9%)	–
Abdominal pain and vomiting	1 (2.6%)	
Cholecystitis	2 (5.3%)	
**Time taken to show symptoms following ultrasound diagnosis**	2 - 5 years	
**Spontaneous resolution**	11 (28.9%)	8 (66.7%)
**Time taken for spontaneous resolution, as shown by ultrasound examination**	3 months to 3 years (Mean: 15 months)	2 months to 5 years (Mean: 9 months)
**Patients remaining with gallbladder abnormalities without symptoms**	17 (44.7)	4 (33.3%)
Surgery	10 (26.3%)	
Video cholecystectomy	3 (30%)	–
Abdominal cholecystectomy	7 (70%)	
**Age at the time of surgery**	9 months to 7 years (Mean: 11 months)	

## DISCUSSION

Ultrasound investigation is considered to be the initial imaging method for diagnostic investigations of the gallbladder and bile duct and presents great sensitivity and specificity for detecting such abnormalities,^16-19^ with an accuracy of up to 96%.^16^

In our study, the prevalence of biliary abnormalities was 9.1% and most patients were non-symptomatic and did not present any known risk factors for gallbladder lithiasis or biliary sludge at the time of the ultrasound diagnosis. 66% of the patients were diagnosed at an age of less than 12 months. Clinical follow-up and serial ultrasound examinations demonstrated that most patients remained without symptoms or had spontaneous resolution of the biliary abnormalities. Considering the absence of risk factors and symptoms, the high frequency in the first year of life and the fact that some cases had spontaneous resolution of the biliary sludge or gallstones, these results are in agreement with the medical literature.^5,7,20,21^ It has been reported^9^ that biliary sludge can be a precursor for biliary lithiasis, but this was not observed in our study.

In recent years, abdominal ultrasound has been widely used in clinics for neonates and children, particularly for detecting cholelithiasis.^4-7^ Clinical conditions such as biliary stasis, parenteral nutrition and prolonged fasting are considered to be high risk factors for development of biliary sludge and calculi, in comparison with hemolytic disease. However, the majority of cases of biliary abnormalities in children, particularly during the neonatal period, are non-symptomatic and idiopathic, with predominance among males and spontaneous resolution within six months after the ultrasound diagnosis in most cases.^9,11,21^

Despite the existence of studies demonstrating increased frequency of cholelithiasis among neonates and children who present associations with particular risk factors,^4-6^ few of them describe associations with DS.^7,12,13,15^ In the general pediatric population, cholelithiasis prevalence has been found to be 0.13% to 0.22%.^1,11^ Case series relating to children with DS have demonstrated high prevalence.^15,22^ In 1986, among 187 DS patients, Buchin et al.^22^ identified only one individual (0.5%) with cholelithiasis, who was 18 years old. Subsequently, in 1993, Llerena et al.^15^ described a group of 145 patients with a 9% prevalence of biliary abnormalities: three children with biliary sludge and ten with gallstones. Neither Buchin et al.^22^ nor Llerena et al.^15^ made any reference to the patients’ ages. In 2001, Toscano et al.^7^ presented six cases (4.7%) cholelithiasis among 126 DS children aged between six months and six years.

The present study confirmed our previous findings^15^ regarding the high prevalence of biliary abnormalities in DS patients. The pathological mechanism for this higher prevalence in DS patients than in the general pediatric population remains unknown, but it could be related to hypercholesterolemia during intrauterine life, as suggested by Bocconi et al. in 1997.^23^

In our series, the great age range seen at the time of the ultrasound investigation was due to two main reasons. Firstly, our hospital is a reference center for the State of Rio de Janeiro^24^ and therefore receives DS patients from different regions of the state at different ages. Secondly, few DS patients were born at our hospital. In such cases, the referral for ultrasound investigation was done while the child was still at the neonatal stage.

Surgical treatment was performed on ten patients (26.3%): six with and four without symptoms. At the beginning of the 1990s, although medical reports demonstrated the possibility of spontaneous resolution of cholelithiasis during the neonatal period,^4^ little was known about the natural history of cholelithiasis in DS patients. At that time, patients with biliary lithiasis that did not spontaneously resolve over the next six months received indication for surgical treatment, even if they did not present any symptoms.^4,25^ In our hospital, ultrasound and clinical follow-up demonstrated that most patients remained free of symptoms and, in many cases, spontaneous resolution of the gallstone or biliary sludge occurred. In the light of this experience, the management of cases of gallstones or biliary sludge, especially regarding surgical procedures, was reviewed. Following this review, the management method changed such that only symptomatic children with abdominal pain, vomiting and/or cholecystitis symptoms were indicated for surgical procedures. The non-symptomatic cases were followed by clinical means and serial abdominal ultrasound examinations. This clinical approach and ultrasound follow-up for DS patients with gallstones or biliary sludge has also been reviewed by other authors.^6,7,9^

## CONCLUSION

Analysis and comparison of our ultrasound data on 547 DS patients in relation to the literature showed that DS must be considered to be a risk factor for the development of biliary lithiasis and biliary sludge in children, especially in the neonatal period without any other associated risk factor. In most DS cases, biliary lithiasis and biliary sludge were non-symptomatic.

It could be concluded that gallstones and biliary sludge in DS patients mostly had favorable evolution and a good prognosis, with spontaneous resolution and non-symptomatic presentation in most patients. These DS patients need to be followed up with serial abdominal ultrasound examinations, and surgical treatment should only be indicated in symptomatic cases or in the presence of cholecystitis.

Further studies are needed, in order to investigate the physiopathological mechanisms and high prevalence of these biliary abnormalities in DS patients.

## Footnotes

* Note: John L.H. Down was a physician in London (1828-1896).
